# Impact of Gambling on the Internet on Middle-Term and Long-Term Recovery from Gambling Disorder: A 2-Year Longitudinal Study

**DOI:** 10.1007/s10899-024-10328-0

**Published:** 2024-09-10

**Authors:** Gaëlle Challet-Bouju, Julie Caillon, Juliette Leboucher, Elsa Thiabaud, Anaïs Saillard, Marianne Balem, Marie Grall-Bronnec

**Affiliations:** 1 Nantes Université, CHU Nantes, UIC Psychiatrie et Santé Mentale, Nantes, F-44000 France; 2 Nantes Université, Univ Tours, CHU Nantes, CHU Tours, INSERM, MethodS in Patients centered outcomes and HEalth ResEarch, SPHERE, Nantes, F-44000 France

**Keywords:** Gambling disorder, Online gambling, Treatment, Recovery, Gambling medium

## Abstract

Online gamblers are more likely to experience gambling problems. The main objective was to compare the frequency of recovery one (middle-term) and two (long-term) years after treatment initiation, according to the gambling medium (Internet vs. land-based gambling). The secondary objectives were (i) to compare online and offline gamblers at inclusion and (ii) to investigate whether the gambling medium was a predictive factor of recovery. Outpatients beginning treatment for a GD (*n* = 237) were assessed at inclusion (treatment initiation) and after 1 and 2 years. Bivariate analyses were performed to compare online and offline gamblers at inclusion and on the frequency of recovery at one and two years. Two multivariate logistic regressions were then performed to identify factors associated with middle- and long-term recovery. The majority of patients achieved middle (74.2%) and long-term (78.9%) recovery, with no difference between online and offline gamblers. The gambling medium was not a predictive factor of recovery. Patients with a higher perceived self-efficacy (OR = 1.04 [1.01–1.07], *p* = .046) and having no history of mood disorders (OR = 11.18 [2.53–49.50], *p* < .001) at inclusion were more likely to achieve middle-term recovery, while long-term recovery was associated with a lower level of sensation seeking (OR = 0.67 [0.48–0.92], *p* = .015) at treatment initiation. Online gambling did not seem to influence middle- and long-term recovery compared to offline gambling. Enhancement of perceived self-efficacy and treatment of mood disorders, and treatment strategies focused on sensation-seeking may represent helpful care strategies for favouring achievement of middle-term recovery and maintenance of long-term recovery, respectively. ClinicalTrials.gov NCT01248767, date of first registration: November 25, 2010.

## Introduction

Gambling disorder (GD) is characterized by persistent and recurrent problematic gambling behavior leading to clinically significant impairment or distress (American Psychiatry Association, 2013). Despite its prevalence, only about 10% on individuals with GD seek help through treatment or support groups (Hodgins et al., 2011; Slutske, 2006). This low help-seeking rate may be attributed to non-linear and fluctuating course of GD, where individuals often experience intermittent periods of problematic and non-problematic gambling (Nower & Blaszczynski, 2008; Pickering et al., 2020; Slutske, 2006; Slutske et al., 2003). These fluctuations might lead individuals to believe they can manage the problem independently (Hodgins et al., 2011).

Recovery from GD is a critical concept and can be viewed as a process, an endpoint, or a conceptual framework (Nower & Blaszczynski, 2008). It does not necessarily require complete abstinence, as controlled gambling can also be a valid treatment objective (Ladouceur et al., 2009). Therefore, defining recovery solely as the absence of gambling episodes may be too restrictive. A more appropriate definition might be the absence of a GD diagnosis, implying the cessation of clinical distress, may be a more valid framework (Gavriel-Fried & Lev-el, 2021; Grall-Bronnec et al., 2021). This definition differs from remission, which implies that none of the diagnostic criteria for GD are met (i.e. no symptoms present) (American Psychiatry Association, 2013). Thus, a disordered gambler in recovery might still gamble and exhibit some GD symptoms, without reaching the threshold for a full diagnosis, particularly regarding significant clinical impairment or distress (American Psychiatry Association, 2013).

Exploring recovery requires monitoring a sufficiently long-term trajectory of gambling behavior and focusing on individuals who have experienced GD symptoms and clinical distress at some point (Nower et al., 2022; Nower & Blaszczynski, 2022). However, longitudinal studies on the clinical evolution of individuals with GD are scarce (Abbott et al., 2018; Billi et al., 2015; Grall-Bronnec et al., 2021; Mazar et al., 2019; Slutske, 2006; Slutske et al., 2003), especially those based on clinical samples. Those individuals, however, represent those who might benefit most from exploring recovery because of direct clinical utility and highest level of gambling-related harm (Pickering et al., 2020). Thus, these studies are crucial as they provide valuable insights into the recovery process and potential predictors of positive treatment outcomes.

Long-term follow-up studies have shown that the trajectory of recovery in GD can vary widely. Some individuals achieve sustained recovery, while others may relapse multiple times before achieving stable recovery (Abbott et al., 2018; Slutske, 2006). Factors that have been identified as predictive of better long-term outcomes include higher motivation for change, stronger social support, and engagement in treatment (Carlbring et al., 2010; Hodgins et al., 2009). Moreover, comorbid mental health conditions, such as depression and anxiety, can negatively impact the recovery process and increase the likelihood of relapse (Yakovenko & Hodgins, 2018).

The rise of technological innovation has facilitated the global expansion of gambling worldwide, with Internet gambling experiencing particularly rapid growth. Studies have reported higher rates of gambling problems among Internet gamblers compared to land-based (offline) gamblers due to several structural characteristics of Internet gambling (greater availability, anonymity, digital payment methods, higher levels of immersion) (Effertz et al., 2018; Gainsbury, 2015; Griffiths, 2003; Griffiths et al., 2011; McCormack & Griffiths, 2013). A difficulty in studying online gambling lies in the fact that many online gamblers also engage in offline gambling or switch between online and offline gambling (J.-M. Costes et al., 2020; Kairouz et al., 2012; Wardle et al., 2011). For mixed-mode gamblers (i.e. gamblers who play both online and offline), it is difficult to determine if gambling problems are associated with land-based gambling, online gambling, or both. Research has shown that online-exclusive gamblers tend to have a lower risk of harm (Blaszczynski et al., 2016), while mixed-mode gamblers often engage in a wider range of games and exhibit higher gambling severity (Leslie & McGrath, 2023). However, these findings primarily stem from population-based surveys. In clinical settings, mixed-mode gamblers are more frequently encountered as they are more likely to seek treatment, especially compared to online-exclusive gamblers (Blaszczynski et al., 2016).

To our knowledge, no longitudinal studies have examined whether participation in Internet gambling can account as a potential predictor of recovery after treatment initiation. The few longitudinal studies on online gambling have focused on severity of gambling problems or relapse rather than recovery. For instance, a five-year follow-up study found that the favorite gambling medium was not a predictor of relapse (Grall-Bronnec et al., 2021). Another recent longitudinal study reported that engagement in certain forms of online gambling (especially casino/slot) was associated with concurrent gambling severity, such associations being strengthened over time, especially during the COVID-19 pandemic (Wardle & Tipping, 2023).

Our main objective was to compare the frequency of recovery one and two years after treatment initiation among outpatients with GD according to whether they gambled online or not. The primary endpoint was the percentage of patients achieving middle-term and long-term recovery, defined as the absence of a GD diagnosis either one (middle-term) or two (long-term) years after treatment initiation.

The secondary objectives were (i) to compare online (including online-exclusive or mixed-mode gamblers) and offline gamblers on sociodemographic, gambling and clinical characteristics at inclusion and (ii) to investigate whether the gambling medium was, among other factors, a predictive factor of recovery one and two years after treatment initiation.

We hypothesized that the gambling trajectories of online gamblers begin earlier and progress more rapidly through each stage (initiation, gambling problems, and treatment). Previous cluster analysis identified an *“early onset and short course*” cluster, where the Internet was more often the preferred gambling medium (Guillou Landreat et al., 2020). This cluster was characterized by a shorter interval between gambling initiation and help-seeking. We hypothesized that this shorter trajectory might lead online gamblers to seek treatment sooner, when the harm is less severe (Haefeli et al., 2011). Consequently, treatment interventions might be more effective in reducing gambling-related damages, leading to higher recovery rates among online gamblers compared to offline gamblers, and making the gambling medium a predictive factor of recovery.

## Methods

### Participants and Procedure

Data were extracted from the EVALADD (EVALuation of behavioral ADDictions) cohort (ClinicalTrials.gov NCT01248767), which consists of a prospective follow-up of outpatients initializing care for a behavioural addiction in an Addictology Department of a Public Hospital in France. The assessment procedure of the EVALADD cohort includes a standardized clinical evaluation at initialization of care, which is repeated after 6 months, 12 months and then each year as long as the patient agrees to continue participating. Clinical evaluations were conducted by trained research staff. All initial evaluations were conducted in person, and follow-ups could be conducted either in person or by phone, depending on the patient’s availability. In the event that the patient is absent at any of the scheduled follow-up visits, our procedure entails making up to three attempts to contact the patient before confirming their dropout status.

For the present study, we extracted data exclusively from patients beginning treatment for gambling-related problems. We included only patients with GD at inclusion, i.e., meeting at least 4 criteria according to the GD section of the DSM-5, recruited between 2009 and 2016. We used only data related to assessments conducted at inclusion (V0), 12 months (V12) and 24 months (V24).

### Measures

#### Socio-demographic data

We collected information about age, sex, marital status, education level, and employment status.

#### Gambling Characteristics

The diagnosis of GD was established based on the Pathological Gambling section of the DSM-IV (American Psychiatric Association, 2000) and reassessed according to the DSM-5 section for GD (i.e., removal of criterion 8 on illegal acts) (American Psychiatry Association, 2013; Petry et al., 2014). Each assessment utilized a 12-month timeframe to evaluate the presence of GD diagnosis. Recovery was defined as the absence of GD diagnosis (number of DSM5 criteria < 4) at each follow-up (V12 and V24), irrespective of whether the individual had ceased gambling activity or not.

Gambling habits and course were assessed through a structured clinical interview specifically designed for the EVALADD cohort.

The gambling course refers to the age of the first gambling experience, the age when gambling-related problems began, an abstinence experience of at least one month and whether the relatives were aware of the gambling problem. The duration of gambling problems was computed as the difference between the age at treatment beginning and the age at the gambling-related problems began.

Regarding gambling habits, patients were requested to indicate the types of gambling activities they used (pure chance games: lottery, scratch cards, slots and video lotteries; skill and chance bank games: horserace betting, sports betting and blackjack; and skill and chance social games: poker (Bjerg, 2010; Boutin, 2010)), in their lifetime and currently (i.e. within the past 12 months). In France, online gambling was officially legalized in 2010, but the French regulation policy only authorizes four types of gambling online: poker, horse race betting, sports betting and lotteries (including draws, bingo and scratch games). All other forms of online gambling, especially online casino games (online slot machines, online table games except poker), have always been banned in France. Moreover, land-based video lotteries and tables games are not allowed in France outside of casinos. Among all the gambling activities they experienced, patients had to identify their favourite game and their favourite gambling medium (online or offline gambling), even for those who had a mixed-mode gambling activity. Moreover, the two groups of interest (online vs. offline gamblers) were constituted as follows: online gamblers were those who reported having gamble to at least one gambling activity online within the past 12 months, whether or not they also gamble offline, whereas offline gamblers were those who did not report any current online gambling activity at all. Thus, the online group combined online-exclusive and mixed-mode gamblers. This strategy allowed us to explore the impact of gambling online, while not excluding the cases of combined practice (both online and offline) which is the rule rather than the exception among online gamblers in care settings.

Patients also had to indicate the therapeutic goal they would like to achieve and to report on the intensity of gambling-related damage in several domains (social, family, work, mental health and financial), using a Likert scale from “Not at all” (score 0) to “Extremely” (score 5). Patients rated their perceived self-efficacy (i.e., perception of control over the gambling problems) and craving (i.e., desire to gamble) during the past week using an analogue visual scale from 0 (“not at all”) to 100 (“extremely”).

Gambling-related cognition was assessed with the 23-item Gambling Related Cognitions Scale (GRCS) (Grall-Bronnec et al., 2012; Raylu & Oei, 2004), which is a self-report questionnaire that explores five dimensions: interpretative bias, illusion of control, predictive control, gambling-related expectancies and perceived inability to stop gambling.

#### Gambling Treatment

In France, there is currently a lack of clinical practice guidelines adressing GD. While certain countries, such as Singapore, have established guidelines over a decade ago (Ministry of Health of Singapore, 2011), recent efforts globally have emphasized the necessity for developing comprehensive guidelines. For instance, the UK National Research Network on Behavioral Addictions, under the leadership of H. Bowden-Jones, has underscored the importance of creating clinical guidelines for pathological gambling (Bowden-Jones et al., 2022). Additionally, the National Institute for Health and Care Excellence (NICE) is anticipated to publish guidance on the identification, assessment, and management of harmful gambling in July 2024 (NICE, 2024).

In light of the absence of specific guidelines in France, our service has adopted a pragmatic approach consisting of three key stages. Initially, patients undergo comprehensive assessments covering various dimensions, including the evaluation of gambling disorder, its consequences, comorbidities, and personality traits. Subsequently, treatment goals are collaboratively established with the patient, which may include goals related to abstinence or reduction of gambling behaviors. Finally, treatment plans are tailored to each individual using a biopsychosocial approach.

In our center, treatment options for GD consist mainly of outpatient treatment, in individual and group formats. Each patient receives at least supportive psychotherapy, with additional interventions such as psychotropic medications or social interventions provided as needed.

Individual therapeutic management consists of psychosocial interventions focusing on gambling as well as the treatment of potential comorbid psychiatric disorders. There is no formal standardized program of treatment, but the management of patients is rather tailored to the specific needs of each individual, according to his/her therapeutic goal and his/her wishes to engage in treatment (Potenza et al., 2019). Individual therapy is provided by psychiatrists specialized in addictology. Patients usually have one consultation per month during the first year of treatment, and the rhythm and duration of treatment is then adjusted according to the clinical evolution of the patient. In addition to psychiatrist consultations, patients can also benefit from individual appointments with a social worker or a psychologist, depending on each situation.

In addition to individual therapeutic management, all patients are offered the opportunity to participate in CBT group sessions tailored specifically to address GD. The CBT group consists of 10 weekly sessions, grouping together 5 to 10 patients with 2 trained therapists. It follows the program proposed by Ladouceur et al., with some additions (especially a session focused on personal values and commitment) (Ladouceur et al., 2003).

Finally, inpatient treatment can also be exceptionally delivered in cases of high suicidal risk, or severe environment difficulties.

In order to include the treatment intensity, which may *a priori* have a major impact on recovery, and given that duration and frequency of treatment are variable among patients, we extracted from the medical records the number of treatment events (sum of the number of individual psychiatric consultations, social worker, nurse and psychologist appointments, group therapy sessions, and hospitalizations). The number of treatment events was computed at V12 and V24 within the period between inclusion and each follow-up.

#### Psychiatric and Addictive Comorbidities

The Mini International Neuropsychiatric Interview (MINI) – version 5.0 (Lecrubier et al., 1997) was used to assess the main axis-I psychiatric disorders, including the investigation of a history of anxiety, mood and addictive disorders, as well as a current risk of suicide.

We also screened patients for the presence of an attention deficit hyperactivity disorder (ADHD) using the Wender-Utah Rating Scale-Child (WURS-C) (Caci et al., 2010; Ward et al., 1993), which enables a retrospective assessment of ADHD in childhood. Patients with a positive screening at the WURS-C were identified as having a “probable ADHD history”. Unlike for other psychiatric or addictive disorders, a formal diagnosis interview was not conducted to confirm definitive diagnosis of ADHD.

#### Life Events

The Questionnaire of Life Events (EVE) (Ferreri et al., 1987) consists of 37 items representing the most frequent life events in 5 categories (family, professional life, social life, marital and emotional life, health). We used a revised version of the EVE questionnaire, including 3 additional items to explore physical or sexual abuse (Guillou-Landreat et al., 2016; Montourcy et al., 2018). We computed a total score as the sum of the intensity of trauma, rated from 0 to 10, experienced for each of the 40 items.

#### Personality Profile

The short 125-item version of the Temperament and Character Inventory (TCI) was used to assess personality traits (Chakroun-Vinciguerra et al., 2005; Cloninger, 1992). It measures seven dimensions through four temperaments (Novelty Seeking, Harm Avoidance, Reward Dependence and Persistence) and three characters (Self-Directedness, Cooperation and Self-Transcendence).

We used the UPPS Impulsive Behaviour Scale (Van der Linden et al., 2006; Whiteside et al., 2005) to measure impulsivity. During data collection, the original UPPS in 45 items and 4 dimensions was replaced by the UPPS-P short version, which includes 20 items and a supplementary dimension (Billieux et al., 2012; Cyders et al., 2014). To compute UPPS-P scores among all patients included, we reconstructed the four available UPPS-P scores (Urgency, lack of Premeditation, lack of Perseverance and Sensation Seeking) based on the responses to the original UPPS for the first patients.

### Statistical Analysis

#### Impact of follow-up Dropout

A sensitivity analysis was performed to estimate whether follow-up dropout may have impacted the results, using Chi-squared tests for qualitative variables and Student’s t-tests for quantitative variables. *P* values were corrected for multiple testing with Benjamini Hocheberg correction.

#### Comparison of Online and Offline Gamblers at Inclusion

Patients were classified into the two groups of interest (online vs. offline gamblers) at inclusion. They were described and compared on all the variables of interest at inclusion and on the frequency of recovery at V12 and V24 using Chi-squared test and Student’s t-tests. *P* values were also corrected for multiple testing.

#### Factors Associated with Gambling Disorder Recovery at 12 and 24 Months

Factors at inclusion associated with recovery at one or two years were identified using two-step multivariate logistic regressions. All the variables of interest were first compared between patients either achieving or not recovery, using bivariate logistic regressions. Then, only variables that were significant at 0.20 were entered into the model, excluding those for which conditions of independence or collinearity were not verified. Finally, an optimization procedure was applied using backward selection with the Akaike information criterion (AIC). At the end, only variables that were significant at a *P* < .05 were interpretable. Due to the expected loss of follow-up in such a long-lasting study, the two multivariate logistic regressions are likely to be underpowered. Thus, we conducted a Monte Carlo simulation analysis to estimate the power associated with each significant p-values obtained in the final models.

All analyses were performed using R 4.0.2 statistical software.

### Ethics

The participants gave their written informed consent prior to their inclusion in the study. This study was approved by the local ethics committee (Groupe Nantais d’Ethique dans le Domaine de la Santé, GNEDS, Nantes).

## Results

### Description of the Sample

**Fig. 1:**
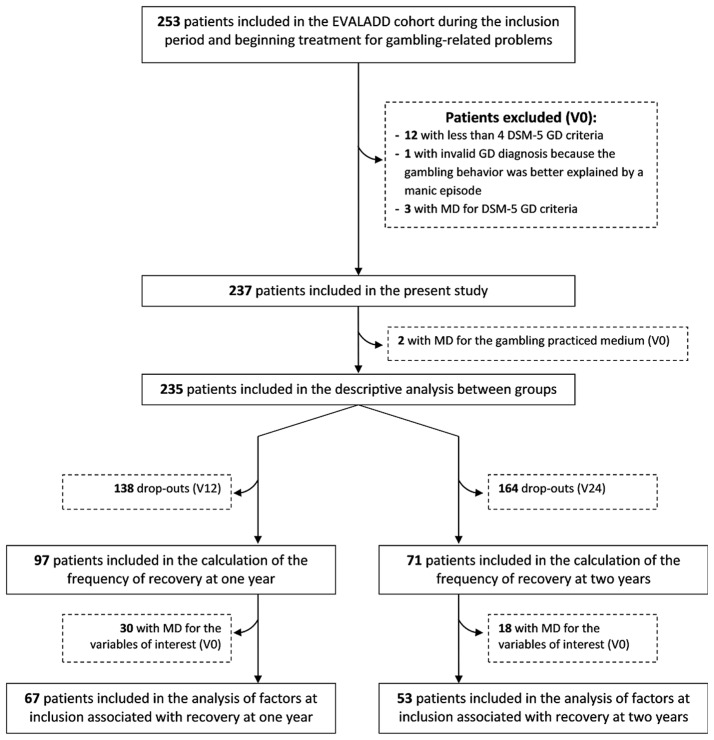
Flow chart of patient selection and inclusion. MD = missing data, V0 = inclusion assessment, V12 = follow-up assessment at 12 months, V24 = follow-up assessment at 24 months

As explained in the flowchart (Fig. 1), of the 237 patients included, 235 had available data to determine the gambling medium at inclusion and were then included in the descriptive analysis.

The global sample was composed of 139 offline (59.1%) and 96 online (40.9%) gamblers. Among online gamblers, a large majority (94.8%) had a combined practice (i.e., gambled both online and offline). The favorite types of gambling were mainly skill and chance bank games (46.8%), followed by pure chance games (37.9%) and then skill and chance social games (14.9%).

A large majority of patients (84.3%) received individual therapeutic management, with the majority of treatment events being consultations with their psychiatrist (88.9%, compared to 4.7% with a nurse, 3.7% with a social worker and 2.7% with a psychologist). Additionally, approximately one third of patients (37.0%) participated in CBT group therapy, while only 5 patients (2.1%) required inpatient treatment.

### Impact of Follow-up Dropout

As detailed in Table 1, the proportion of patients dropping out at each assessment was similar between online and offline gamblers, reaching approximately 60% for V12 and 70% for V24. Moreover, Table 2 indicates that, compared to patients included in the follow-up, those who dropped out at V12 were slightly younger (39.6 vs. 45.5 years, *p* < .001). Moreover, those who dropped out at V12 or V24 received less care (4.5 versus 11.7 treatment events at V12 and 5.7 versus 16.1 at V24, *p* < .001), which is consistent with the loss of follow-up. Finally, there was no difference between patients who dropped out or not regarding the severity of gambling disorder at inclusion.

**Table 1:** Sample size of each group at each assessment

	Total		Offline (at V0)		Online (at V0)	
	n	% ^a^	n	% ^a^	n	% ^a^
V0	235	100.0%	139	100.0%	96	100.0%
V12	97	41.3%	67	48.2%	30	31.2%
V24	71	30.2%	50	36.0%	21	21.9%

V0 = inclusion assessment; V12 = follow-up assessment at 12 months; V24 = follow-up assessment at 24 months

^a^ Percentages indicate the proportion of patients remaining for each assessment from all patients included

**Table 2:** Sensibility analysis: comparison of patients included in each follow-up with patients that dropped out, on characteristics at inclusion (*n* = 235)

		One-year follow-up (V12)					Two-year follow-up (V24)				
		n	Patients included	n	Patients dropped out	Adjusted p-value ^d^	n	Patients included	n	Patients dropped out	Adjusted p-value ^d^
Sociodemographic characteristics											
	Age (µ (σ))	97	45.49 (12.87)	138	39.58 (12.54)	**< 0.001**	71	44.65 (12.79)	164	40.88 (12.94)	0.16
	Sex – women (n (%))	97	22 (22.68%)	138	15 (10.87%)	0.12	71	16 (22.54%)	164	21 (12.80%)	0.24
	Education level ^a^ (µ (σ))	97	11.60 (3.13)	137	11.37 (3.87)	0.93	71	11.93 (2.83)	163	11.26 (3.85)	0.55
	Marital status – living alone (n (%))	97	45 (46.39%)	138	61 (44.20%)	0.93	71	33 (46.48%)	164	73 (44.51%)	0.93
	Professional activity – being inactive (n (%))	96	38 (39.58%)	137	45 (32.85%)	0.73	70	25 (35.71%)	163	58 (35.58%)	1.00
Psychiatric and addictive comorbidities											
	History of mood disorders (n (%))	97	45 (46.39%)	138	84 (60.87%)	0.14	71	36 (50.70%)	164	93 (56.71%)	0.82
	History of anxiety disorders (n (%))	97	41 (42.27%)	138	68 (49.28%)	0.73	71	35 (49.30%)	164	74 (45.12%)	0.92
	History of addictive disorders (n (%))	97	37 (38.14%)	138	74 (53.62%)	0.13	71	25 (35.21%)	164	86 (52.44%)	0.12
	Probable history of ADHD (n (%))	96	26 (27.08%)	128	38 (29.69%)	0.92	70	20 (28.57%)	154	44 (28.57)	1.00
	Current suicidal risk (n (%))	97	48 (49.48%)	138	48 (34.78%)	0.13	71	36 (50.70%)	164	60 (36.59%)	0.18
Gambling characteristics											
	Age of first gambling experience (µ (σ))	96	18.94 (8.89)	135	18.39 (9.15)	0.92	70	18.53 (7.98)	161	18.65 (9.46)	0.99
	Duration of gambling problems (µ (σ))	96	9.10 (8.61)	135	6.33 (6.41)	0.08	70	9.17 (7.92)	161	6.75 (7.23)	0.13
	Gambling medium - offline (n (%))	97	67 (69.07%)	138	72 (52.17%)	0.10	71	50 (70.42%)	164	89 (54.27%)	0.13
	Preferred gambling activity (n (%)) Pure chance games Skill and chance bank games Skill and chance social games	96	38 (39.58%) 48 (50.00%) 10 (10.42%)	138	51 (36.96%) 62 (44.93%) 25 (18.12%)	0.77	70	30 (42.86%) 34 (48.57%) 6 (8.57%)	164	59 (35.98%) 76 (46.34%) 29 (17.68%)	0.55
	Relatives aware of the gambling problem (n (%))	97	91 (93.81%)	138	129 (93.48%)	0.99	71	68 (95.77%)	164	152 (92.68%)	0.82
	Experience of abstinence of at least one month (n (%))	96	64 (66.67%)	136	85 (62.50%)	0.91	70	46 (65.71%)	162	103 (63.58%)	0.93
	Craving [/100] (µ (σ))	97	48.61 (33.33)	138	54.89 (32.11)	0.47	71	45.49 (33.29)	164	55.24 (32.09)	0.17
	Perceived self-efficacy [/100] (µ (σ))	97	37.58 (29.61)	138	40.04 (30.68)	0.92	71	38.38 (28.98)	164	40.00 (30.80)	0.96
	Severity of gambling disorders ^b^ (µ (σ))	97	6.56 (1.54)	138	6.66 (1.51)	0.92	71	6.72 (1.60)	164	6.57 (1.49)	0.91
	Intensity of gambling-related damages [/25] (µ (σ))	95	13.42 (5.13)	135	13.79 (5.79)	0.96	70	13.60 (5.27)	160	13.47 (5.64)	0.98
Gambling treatment											
	Therapeutic goal (n (%)) To achieve abstinence To reduce/control gambling Do not know/no objective	96	54 (56.25%) 38 (39.58%) 4 (4.17%)	132	67 (50.76%) 59 (44.70%) 6 (4.55%)	0.93	70	44 (62.86%) 25 (35.71%) 1 (1.43%)	158	77 (48.73%) 72 (45.57%) 9 (5.70%)	0.31
	Number of any kind of treatment events V12 ^c^ (µ (σ))	97	11.65 (8.60)	138	4.49 (6.57)	**< 0.001**	71	11.92 (8.81)	164	5.51 (7.20)	**< 0.001**
	Number of any kind of treatment events V24 ^c^ (µ (σ))	97	14.99 (10.35)	138	4.49 (6.58)	**< 0.001**	71	16.07 (10.73)	164	5.69 (7.46)	**< 0.001**
Gambling-related cognitive distortions											
	GRCS_GE [/28] (µ (σ))	69	15.86 (4.97)	99	15.46 (6.08)	0.92	55	15.80 (5.38)	113	15.54 (5.78)	0.93
	GRCS_IC [/28] (µ (σ))	69	8.77 (4.80)	99	8.07 (5.07)	0.82	55	8.27 (4.36)	113	8.40 (5.24)	0.98
	GRCS_PC [/42] (µ (σ))	69	20.84 (7.02)	99	19.08 (7.28)	0.40	55	20.49 (7.43)	113	19.47 (7.11)	0.82
	GRCS_IS [/35] (µ (σ))	69	24.65 (7.06)	99	23.97 (6.90)	0.92	55	24.60 (7.22)	113	24.08 (6.85)	0.92
	GRCS_IB [/28] (µ (σ))	69	16.32 (5.63)	99	16.19 (6.18)	0.98	55	15.73 (5.76)	113	16.50 (6.05)	0.83
Impulsivity											
	UPPS_U [/16] (µ (σ))	95	10.95 (2.89)	128	11.09 (2.85)	0.93	70	11.09 (2.89)	153	10.99 (2.86)	0.96
	UPPS_LPrem [/16] (µ (σ))	95	8.96 (2.66)	128	9.08 (2.34)	0.93	70	9.10 (2.72)	153	8.99 (2.37)	0.93
	UPPS_LPers [/16] (µ (σ))	95	8.34 (2.37)	128	8.11 (2.48)	0.91	70	8.43 (2.34)	153	8.10 (2.47)	0.82
	UPPS_SS [/16] (µ (σ))	95	9.96 (2.93)	128	10.30 (3.03)	0.82	70	10.01 (2.75)	153	10.22 (3.10)	0.92
Life events											
	EVE [/400] (µ (σ))	96	28.07 (41.92)	136	23.65 (40.31)	0.82	70	32.71 (44.54)	162	22.35 (39.03)	0.29
Personality											
	TCI_NS [/100] (µ (σ))	95	57.53 (17.44)	127	63.11 (16.88)	0.12	70	56.36 (17.21)	152	62.73 (17.03)	0.10
	TCI_HA [/100] (µ (σ))	95	54.16 (25.63)	127	50.59 (22.88)	0.73	70	54.07 (28.04)	152	51.22 (22.10)	0.82
	TCI_RD [/100] (µ (σ))	90	55.19 (18.31)	122	56.38 (16.46)	0.92	66	55.86 (16.96)	146	55.88 (17.42)	1.00
	TCI_P [/100] (µ (σ))	95	49.47 (30.01)	127	56.22 (31.34)	0.38	70	53.43 (29.04)	152	53.29 (31.81)	1.00
	TCI_SD [/100] (µ (σ))	95	56.42 (20.48)	127	56.42 (20.48)	1.00	70	57.26 (20.95)	152	55.89 (20.19)	0.92
	TCI_C [/100] (µ (σ))	95	71.75 (15.41)	127	70.82 (16.48)	0.92	70	71.94 (16.39)	152	70.88 (15.86)	0.92
	TCI_ST [/100] (µ (σ))	91	30.22 (22.09)	118	32.81 (22.09)	0.82	67	28.70 (22.92)	142	33.08 (21.60)	0.55

ADHD = Attention Deficit Hyperactivity Disorder; GRCS = Gambling Related Cognition Scale; GRCS_GE = Gambling-related Expectancies dimension of the GRCS; GRCS_IC = Illusion of Control dimension of the GRCS; GRCS_PC = Predictive Control dimension of the GRCS; GRCS_IS = perceived Inability to Stop dimension of the GRCS; GRCS_IB = Interpretative control/Bias dimension of the GRCS; UPPS = UPPS Impulsive Behaviour scale; UPPS_U = Urgency dimension of the UPPS; UPPS_LPrem = Lack of Premeditation dimension of the UPPS; UPPS_LPers = Lack of Perseverance dimension of the UPPS; UPPS_SS = Sensation Seeking dimension of the UPPS; EVE = Questionnaire of Life Events (total score); TCI = Temperament and Character Inventory (125 items version); TCI_NS = Novelty Seeking dimension of the TCI; TCI_HA = Harm Avoidance dimension of the TCI; TCI_RD = Reward Dependence dimension of the TCI; TCI_P = Persistence dimension of the TCI; TCI_SD = Self-Directedness dimension of the TCI; TCI_C = Cooperation dimension of the TCI; TCI_ST = Self-Transcendence dimension of the TCI

^a^ Education level = Number of completed years of education

^b^ Severity of gambling disorders = number of positive DSM5 criteria (0 to 9)

^c^ Number of any kind of treatment events = number of treatment events (individual consultation, group session, hospitalization), within the period between inclusion and the corresponding follow-up (V12 or V24). This is the only variable that is not strictly collected at inclusion

^d^*P* values indicated in bold are those under 0.05 (level of statistical significance)

### Comparison of Online and Offline Gamblers at Inclusion

The description and comparative analysis of online versus offline gamblers are presented in Table 3.

**Table 3:** Description and comparison of patients between the two groups (online vs. offline) on characteristics at inclusion (*n* = 235)

		*n*	Offline *N* = 139	Online *N* = 96		Adjusted *p*-value ^d^
Sociodemographic characteristics						
	Age (µ (σ))	235	46.65 (12.34)	35.32 (10.84)	**< 0.001**	
	Sex – women (n (%))	235	27 (19.42%)	10 (10.42%)	0.17	
	Education level ^a^ (µ (σ))	234	11.04 (3.79)	12.08 (3.16)	0.12	
	Marital status – living alone (n (%))	235	62 (44.60%)	44 (45.83%)	0.89	
	Professional activity – being inactive (n (%))	233	54 (39.42%)	29 (30.21%)	0.29	
Gambling characteristics						
	Age of first gambling experience (µ (σ))	231	20.99 (9.77)	15.16 (6.44)	**< 0.001**	
	Duration of gambling problems (µ (σ))	231	8.99 (8.71)	5.25 (4.41)	**< 0.001**	
	Preferred gambling activity (n (%)) Pure chance games Skill and chance bank games Skill and chance social games	234	63 (45.65%) 69 (50.00%) 6 (4.35%)	26 (27.08%) 41 (42.71%) 29 (30.21%)	**< 0.001**	
	Relatives aware of the gambling problem (n (%))	235	126 (90.65%)	94 (97.92%)	0.12	
	Experience of abstinence of at least one month (n (%))	235	88 (63.77%)	61 (64.89%)	0.89	
	Craving [/100] (µ (σ))	235	49.86 (33.45)	55.83 (31.41)	0.31	
	Perceived self-efficacy [/100] (µ (σ))	235	39.35 (30.38)	38.54 (30.10)	0.89	
	Severity of gambling disorders ^b^ (µ (σ))	235	6.47 (1.62)	6.83 (1.34)	0.18	
	Intensity of gambling-related damages [/25] (µ (σ))	235	13.05 (5.62)	14.18 (5.32)	0.29	
Gambling treatment						
	Therapeutic goal (n (%)) To achieve abstinence To reduce/control gambling Do not know/no objective	228	79 (58.09%) 52 (38.24%) 5 (3.68%)	42 (45.65%) 45 (48.91%) 5 (5.43%)	0.31	
	Number of any kind of treatment events V12 ^c^ (µ (σ))	235	7.72 (8.27)	7.04 (8.24)	0.68	
	Number of any kind of treatment events V24 ^c^ (µ (σ))	235	9.19 (9.87)	8.30 (9.73)	0.65	
Gambling-related cognitive distortions						
	GRCS_GE [/28] (µ (σ))	169	16.07 (5.77)	14.92 (5.38)	0.31	
	GRCS_IC [/28] (µ (σ))	169	8.96 (5.49)	7.40 (3.82)	0.17	
	GRCS_PC [/42] (µ (σ))	169	20.76 (7.74)	18.29 (6.03)	0.12	
	GRCS_IS [/35] (µ (σ))	169	25.07 (6.66)	22.96 (7.27)	0.17	
	GRCS_IB [/28] (µ (σ))	169	16.40 (6.05)	16.00 (5.82)	0.67	
Psychiatric and addictive comorbidities						
	History of mood disorders (n (%))	235	77 (55.40%)	52 (54.17%)	0.89	
	History of anxiety disorders (n (%))	235	44 (45.83%)	65 (46.76%)	0.89	
	History of addictive disorders (n (%))	235	57 (41.01%)	54 (56.25%)	0.12	
	Probable history of ADHD (n (%))	224	41 (30.60%)	23 (25.56%)	0.60	
	Current suicidal risk (n (%))	235	64 (46.04%)	32 (33.33%)	0.17	
Impulsivity						
	UPPS_U [/16] (µ (σ))	223	11.26 (2.94)	10.68 (2.72)	0.29	
	UPPS_LPrem [/16] (µ (σ))	223	8.93 (2.74)	9.17 (2.03)	0.65	
	UPPS_LPers [/16] (µ (σ))	223	8.03 (2.54)	2.47 (2.25)	0.31	
	UPPS_SS [/16] (µ (σ))	223	8.03 (2.54)	8.47 (2.25)	0.31	
Life events						
	EVE [/400] (µ (σ))	232	30.51 (44.90)	18.23 (33.37)	0.12	
Personality						
	TCI_NS [/100] (µ (σ))	222	59.66 (16.88)	62.30 (17.90)	0.40	
	TCI_HA [/100] (µ (σ))	222	50.19 (24.79)	55.00 (22.88)	0.29	
	TCI_RD [/100] (µ (σ))	222	55.16 (16.68)	56.96 (18.11)	0.64	
	TCI_P [/100] (µ (σ))	222	56.24 (30.17)	48.99 (31.62)	0.21	
	TCI_SD [/100] (µ (σ))	222	56.50 (21.04)	56.04 (19.52)	0.89	
	TCI_C [/100] (µ (σ))	222	70.78 (16.70)	71.87 (14.97)	0.76	
	TCI_ST [/100] (µ (σ))	209	34.61 (21.96)	27.23 (21.62)	0.12	

ADHD = Attention Deficit Hyperactivity Disorder; GRCS = Gambling Related Cognition Scale; GRCS_GE = Gambling-related Expectancies dimension of the GRCS; GRCS_IC = Illusion of Control dimension of the GRCS; GRCS_PC = Predictive Control dimension of the GRCS; GRCS_IS = perceived Inability to Stop dimension of the GRCS; GRCS_IB = Interpretative control/Bias dimension of the GRCS; UPPS = UPPS Impulsive Behaviour scale; UPPS_U = Urgency dimension of the UPPS; UPPS_LPrem = Lack of Premeditation dimension of the UPPS; UPPS_LPers = Lack of Perseverance dimension of the UPPS; UPPS_SS = Sensation Seeking dimension of the UPPS; EVE = Questionnaire of Life Events (total score); TCI = Temperament and Character Inventory (125 items version); TCI_NS = Novelty Seeking dimension of the TCI; TCI_HA = Harm Avoidance dimension of the TCI; TCI_RD = Reward Dependence dimension of the TCI; TCI_P = Persistence dimension of the TCI; TCI_SD = Self-Directedness dimension of the TCI; TCI_C = Cooperation dimension of the TCI; TCI_ST = Self-Transcendence dimension of the TCI

^a^ Education level = Number of completed years of education

^b^ Severity of gambling disorders = number of positive DSM5 criteria (0 to 9)

^c^ Number of any kind of treatment events = number of treatment events (individual consultation, group session, hospitalization), within the period between inclusion and the corresponding follow-up (V12 or V24). This is the only variable that is not strictly collected at inclusion

^d^*P* values indicated in bold are those under 0.05 (level of statistical significance)

#### Socio-demographic data

Both online and offline gamblers were mainly men (89.6% and 80.6% respectively). Online gamblers were approximately 11 years younger than offline gamblers (35.3 versus 46.7 years, *p* < .001). Education level, marital status and professional activity did not differ between online and offline gamblers.

#### Gambling Characteristics and gambling-related Cognitive Distortions

Online gamblers were initiated earlier than offline gamblers at an age (15 years old) under the legal age for gambling in France (18 years old) (15.2 versus 21.0 years, *p* < .001). The duration of gambling problems before the beginning of treatment was shorter for online gamblers, being approximately half of that of offline gamblers (5.3 versus 9.0 years, *p* < .001). This means that the global gambling course was earlier and quicker for online gamblers at all steps of the process (gambling initiation - start of gambling problems - treatment beginning).

Compared to offline gamblers, online gamblers preferred gambling activities involving skill, especially poker (30.2% versus 4.4% for poker, *p* < .001).

The large majority of patients (93.6%) had informed their families of their gambling problems, and close to two-thirds (63.4%) of the sample had experienced a gambling abstinence of at least one month, with no difference between online and offline gamblers.

Perceived self-efficacy, craving, severity of the gambling disorder, intensity of gambling-related damages and levels of gambling-related cognition did not differ between online and offline gamblers.

#### Gambling Treatment

The therapeutic goal at inclusion was similar for online and offline gamblers, i.e., there was almost an equal distribution between the objectives of achieving abstinence (53.1%) and reducing/controlling gambling (42.5%).

The number of treatment events was also similar between online and offline gamblers, either at V12 or V24.

#### Clinical Profiles

The frequency of psychiatric and addictive comorbidities, the intensity of negative life events, the impulsivity profile and the personality profile did not differ between online and offline gamblers.

#### Frequency of Gambling Disorder Recovery at 12 and 24 Months

The final sample used for calculation of frequency of recovery was composed of 97 (V12) and 71 (V24) patients, due to loss of follow-up (see Fig. 1). The majority of patients with available data achieved recovery at one year (74.2%) and two years (78.9%) after treatment initiation. As detailed in Table 4, the observed frequencies of recovery seem to be slightly higher for the offline group, although the difference did not reach significance.

**Table 4:** Frequency of recovery at 12 and 24 months according to the gambling medium (*n* = 168)

		Online		Offline		*P* value
		n	%	n	%	
Recovery at one year (*n* = 97)						0.52
	Yes	21	70.0%	51	76.1%	
	No	9	30.0%	16	23.9%	
Recovery at two years (*n* = 71)						0.31
	Yes	15	71.4%	41	82.0%	
	No	6	28.6%	9	18.0%	

### Factors associated with gambling disorder recovery at 12 and 24 months – place of the gambling medium

The sample used for the identification of factors associated with recovery at one year and two years was composed of 67 (V12) and 53 (V24) patients, due to missing data in variables of interest (see Fig. 1). Among the 38 candidate variables, 23 and 10 variables were retained to be entered in the multivariate logistic regression for the prediction of recovery at one year and two years, respectively. After the optimization selection procedure, we obtained the final models detailed in Tables 5 and 6.

**Table 5:** Factors associated with recovery at one year (*n* = 67)

		OR	CI 95%	*P* value ^a^	Estimated power ^b^
Perceived self-efficacy		1.04	[1.01–1.07]	**0.046**	0.78
Absence of mood disorders		11.18	[2.53–49.50]	**0.001**	1.00
Severity of GD at inclusion		0.67	[0.43–106]	0.08	-

OR = Odds Ratio; IC95 = Confidence Intervals at 95%; GD = Gambling Disorder

^a^*P* values indicated in bold are those under 0.05 (level of statistical significance)

^b^ Estimated power was calculated through a Monte Carlo simulation for significant variables only

**Table 6:** Factors associated with recovery at two years (*n* = 53)

		OR	CI 95%	*P* value ^a^	Estimated power ^b^
Sensation Seeking (UPPS-SS)		0.67	[0.48–0.92]	**0.015**	0.58
Self-Directedness (TCI-SD)		1.04	[1.00–1.08]	0.05	-
Offline gambling		4.67	[0.90-24.21]	0.07	-

OR = Odds Ratio; IC95 = Confidence Intervals at 95%; UPPS_SS = Sensation Seeking dimension of the UPPS Impulsive Behaviour scale; TCI_SD = Self-Directedness dimension of the Temperament and Character Inventory (125 items version)

^a^*P* values indicated in bold are those under 0.05 (level of statistical significance)

^b^ Estimated power was calculated through a Monte Carlo simulation for significant variables only

The number of treatment events (calculated within the period between inclusion and the corresponding follow-up) was not a predictor of recovery, whether at one year or two years. Gamblers with a higher perceived self-efficacy (OR 1.04 [1.01–1.07], *p* = .046) and those with no history of mood disorders (OR 11.18 [2.53–49.50], *p* < .001) at inclusion were more likely to achieve recovery one year after treatment initiation. Moreover, a lower level of sensation seeking (OR 0.67 [0.48–0.92], *p* = .015) at inclusion was a significant predictor of recovery two years after treatment initiation. Within the sample used for identifying factors associated with recovery at two years (*n* = 53), 33 of the 42 patients in recovery (78.6%) were already in recovery at V12. For both analyses, the gambling medium at inclusion was not a predictive factor of recovery. Among online gamblers included in both analyses, only 1.5% (V12, *n* = 1) and 1.4% (V24, *n* = 1) had an online-exclusive practice.

## Discussion

### Main Results

#### Frequency of Recovery in Online Versus Offline Gamblers

The frequencies of middle-term recovery were very similar between online and offline gamblers, and even if the observed frequency of long-term recovery seemed higher in the offline group, the difference did not reach statistical significance. This may be due to a lack of power, especially in the online group (*n* = 21 at V24). It is nevertheless interesting to observe that recovery concerned a large majority (more than three quarters) of the patients included, even two years after treatment initiation. However, we cannot exclude that patients who dropped out were those with poorer clinical outcomes and that the frequency of recovery may be overestimated. Nevertheless, patients who dropped out during the follow-up had a shorter duration of gambling problems and there was no difference between them and those included in the analysis regarding gambling disorder severity at inclusion, which would rather argue for an underestimation of the recovery than the opposite. In any case, it seems important to continue efforts to promote care in specialized hospitals with multidisciplinary help (medical, psychological and social support).

#### Comparison of Online and Offline Gamblers

As we had assumed, online gamblers had an earlier and shorter gambling course than offline gamblers at all steps of the process (gambling initiation - gambling problems - treatment), which was consistent with the previously found *“early onset and short course*” cluster in which the Internet was more often the preferred gambling medium (Guillou Landreat et al., 2020). Earlier gambling initiation has consistently been associated with a higher risk of gambling problems (Dowling et al., 2017; Kessler et al., 2008; Lynch et al., 2004; Sharman et al., 2019), even if the relationship was found to be more related to family-level characteristics (Slutske et al., 2014). In our study, gambling initiation occurred before the legal age for online gamblers. Compared to offline gambling, where the retailer can easily identify people under the legal age, access to online gambling by minors may be easier by using, for example, the online gambling account of an older friend or relative. As argued by Dowling et al., even if gambling products are made available legally only for adults in a majority of countries, youth gambling is common and correlates with a variety of negative consequences (Dowling et al., 2017; Newall et al., 2021). Even more problematic, gambling patterns developed in childhood and adolescence may favour later adult gambling problems (Derevensky et al., 2003; Dowling et al., 2017). We can hypothesize that early access to online gambling for youths may favour future online gambling patterns in adults, with potentially a higher risk of gambling problems (Effertz et al., 2018; Gainsbury, 2015; Griffiths, 2003; Griffiths et al., 2011; McCormack & Griffiths, 2013). Moreover, having experienced violation of the law to achieve the possibility of gambling as a youth in a country where it is forbidden may also favour experiencing illegal gambling as an adult, which has been associated with higher levels of harm (Costes et al., 2016).

#### Factors Associated with Middle-Term and Long-Term Recovery

Contrary to our expectations, the gambling medium does not seem to influence middle-term and long-term recovery from GD after treatment initiation. As the very large majority of online gamblers from our sample were mixed-mode gamblers, almost all gamblers played offline and we cannot exclude that this may have prevented the medium from being identified as a predictive factor of recovery in the models. However, this situation corresponds to the reality of the patient population in our center. The fact that gambling on the Internet in addition to gambling offline does not impede recovery in patients initiating a specialized treatment is quite contrary to what might have been expected. Indeed, previous population-based surveys have shown that mixed-mode problematic gamblers display higher problematic gambling severity and gambling-related harm (Blaszczynski et al., 2016; Hing et al., 2022; Leslie & McGrath, 2023). One possible explanation is that the level of problematic gambling severity is high for all gamblers seeking treatment for a gambling problem, whatever they gamble or not on the internet. This hypothesis is confirmed in our sample, as the severity of gambling problems at inclusion was similar between online and offline gamblers, contrary to what have been reported in population-based surveys (Hing et al., 2022). Moreover, another possible explanation is due to the specific French context, that may be more protective than in other countries. Indeed, in France, online gambling was legalized in 2010, but authorized online gambling forms are limited to lotteries, poker, sports betting and horse race betting. More specifically, casino games (especially electronic gaming machines (EGMs) are still forbidden online in France. EGMs have consistently been associated with higher prevalence of gambling problems (Dowling et al., 2005; Wardle et al., 2011) and have been identified as the most common form associated with gambling related-harm in online-exclusive, offline-exclusive and mixed-mode gamblers (Hing et al., 2022). Even if French gamblers may access online casinos through illegal offshore operators, it was estimated that nearly 2 out of 10 French online gamblers gamble on an offer that is not legal or regulated, including online casino games but also financial betting or e-sport betting) (J. M. Costes & Eroukmanoff, 2018). Thus, we cannot exclude that this specific regulation could have limited the importance of the gambling medium in predicting recovery.

One interesting result is that the factors associated with middle-term and long-term recovery were not the same, even if the large majority of patients who achieved recovery after two years were already in recovery at one year.

Predictive factors of middle-term recovery were a higher level of perceived self-efficacy and having no history of mood disorders. In the framework of gambling, perceived self-efficacy is defined as the confidence of an individual regarding his/her own capacity to avoid involvement in problem gambling (Bozzato et al., 2020). It has been demonstrated as a mediating factor between perceived impacts of gambling advertising and the severity of problem gambling (Quinn et al., 2019). Several studies have demonstrated the fundamental role of perceived self-efficacy in controlling and maintaining control over various addictive behaviours (Casey et al., 2008). The level of perceived self-efficacy is thought to be predictive of future behaviour and to have a high influence in the success of treatment (Hodgins et al., 2004). In a previous study, we established that the perceived inability to stop gambling, which is the opposite of perceived self-efficacy, was a predictor of suicidal risk in patients with a gambling disorder (Guillou-Landreat et al., 2016). The notion of hope is thus a decisive factor supporting recovery. As a consequence, treatment options focused on enhancing perceived self-efficacy may be an effective way to reduce the risk of relapse (Hodgins et al., 2004). Dual disorder (i.e., the co-occurrence of a mental health disorder and an addictive disorder) is a frequent condition in GD (Szerman et al., 2020). Mood disorders represent the most prevalent mental health disorder in individuals with GD (Lorains et al., 2011; Petry et al., 2005). According to the well-accepted self-medication hypothesis, gambling may stimulate the brain reward circuitry to produce calm and relaxation, which may help the individual cope with symptoms of his co-occurring mental health disorder (Szerman et al., 2020) and escape painful emotions (Bilevicius et al., 2018). This was especially developed in the pathway model of problem gambling (Blaszczynski & Nower, 2002; Nower et al., 2022) which identified a subgroup of ‘emotionally vulnerable’ individuals, who primarily gamble to escape aversive mood states. Thus, a history of a mood disorder may reinforce gambling behaviour and make it difficult for gamblers to achieve recovery.

Regarding long-term recovery, only one predictive factor was found significant and was related to a personality trait, namely, a low level of sensation seeking. Sensation-seeking refers to the tendency to engage in and maintain exciting activities and to experiment with new and potentially dangerous activities (Whiteside et al., 2005). Sensation seeking is traditionally associated with motivational factors involved in approach and avoidance behaviours and, more specifically, in reward sensitivity (Billieux et al., 2014). As a consequence, sensation seeking is more related to experimentation of gambling rather than GD itself (Billieux et al., 2014). Sensation seeking was associated with gambling frequency after controlling for shared genetic and environmental influences and was identified as a direct risk factor for gambling (Huggett et al., 2021). Within the theoretical framework of negative recovery capital, sensation seeking has been identified as one factor that impedes individuals from recovering from GD (Gavriel-Fried & Lev-el, 2021). One may assume that pathological gamblers in remission that display high levels of sensation seeking may be more susceptible to experimental lapses of resumption of gambling behaviour because of a higher motivational and reward value attributed to gambling.

One may find it surprising that depression has a very huge effect for achievement of middle-term but not long-term recovery, and that a quite stable personality factor (sensation seeking) is predictive of long-term but not middle-term recovery. One possible explanation is that a large majority of gamblers who were in recovery at two years were already in recovery at one year (78.6%). This means that the factors identified at two years were more related to sustainment rather than achievement of recovery, contrary to the factor identified at one year that was only related to achievement of recovery. This may explain why the predictive factors identified were not the same at both time points. Another explanation is that depression was taken into account as a predictive factor based on measurement at inclusion. Indeed, if there was 54.0% of patients with depression at inclusion, they were only 16.9% at V24, indicating that the majority of depressive disorders were resolved during the two-year follow-up. Thus, having a depressive disorder at the beginning of care may impede the achievement of recovery in the earlier stages of gambling treatment, but a lesser effect further away from treatment initiation on sustained recovery due to appropriate treatment that occurred in the meantime.

### Limitations and Strengths

Several limitations of this work must be highlighted. First, the sample used for identifying predictive factors of recovery was quite small due to the loss of follow-up, especially in the online group, which could have led to a lack of power. This could be one explanation why the number of treatment events did not appear to predict recovery, contrary to what might have been expected. This may have smoothed the differences in the number of treatment events between those in recovery or not. Despite the low sample size, the power analysis conducted for the significant variables found in the two models revealed that the power was high for the one-year model (0.78 and 1.00), whereas it was more moderated for the two-year model (0.58). As a consequence, the results related to the two-year analysis should be taken with caution. Moreover, the high drop-out rate (59% at one year and 70% at two years) may also have biased the results, as the longitudinal analysis of predictors of recovery was performed only on patients with available data. However, high drop-out rates are commonly encountered in long-term follow-up studies on gambling, especially when participation in follow-ups does not give rise to a financial compensation, as in our study: e.g. 122 of 353 individuals remained in the study during a 5-year treatment follow-up (drop-out rate: 66%) (Kruse-Diehr et al., 2022), 274 of 575 completed the 2-year follow-up in a 3-year longitudinal classification study (drop-out rate: 52%) (Chamberlain et al., 2020). Second, certain factors that may contribute to the maintenance of GD, such as emotional distress (Granero et al., 2020), were not included in our study. Finally, we choose to include among online gamblers both online-exclusive and mixed-mode gamblers, contrary to recent literature that recommend assessing them separately (Hing et al., 2022). As we were in a clinical setting, where mixed-mode gamblers are overwhelmingly dominant among online gamblers, we were not able to isolate a third group of online-exclusive gamblers. However, online-exclusive gamblers represented only a very few proportion of online gamblers in the models predicting recovery, so that the results may not have been biased by this choice.

Despite these limitations, this work is an important advance in GD research because the issue of recovery from GD has received little attention in the literature. This is a longitudinal study that provides a better understanding of the differences related to the gambling medium and of the predictors of middle-term and long-term recovery. Finally, patients included in the follow-up were highly similar to those who dropped out, including factors that could have impacted recovery, such as the therapeutic goal, the presence of psychiatric comorbidities, GD severity, gambling-related damage, the level of perceived self-efficacy or the awareness of relatives. As a consequence, loss of follow-up did not seem to bias the results.

### Conclusions and Perspectives

The results suggested that the gambling medium does not influence middle-term and long-term recovery from GD. Nevertheless, the earlier and shorter gambling course of online gamblers must be taken into account to increase prevention among the youngest, inform families on the risks of gambling, especially on the internet, and promote access to care (Hubert & Griffiths, 2018). As it may be easier to bypass the ban for gambling for minors on online compared to offline gambling, the protection of minors must be accompanied by more information and prevention messages.

At 1 and 2 years after treatment initiation, we observed recovery in the large majority of patients. The enhancement of perceived self-efficacy by motivational interviewing and cognitive therapy, and the treatment mood disorders by specific medications may represent helpful care strategies for favouring the achievement of middle-term recovery. Moreover, treatment strategies focused on the management of sensation seeking may be beneficial for maintaining long-term recovery. Finally, as the frequency of recovery was stable or even increased between one and two years after treatment initiation and because factors associated with middle- and long-term recovery were not the same, it is of high importance to encourage long-term follow-up, even after remission, to help patients maintain recovery and prevent relapses.

## Data Availability

The data sets generated and analyzed during the current study are personal data under the responsibility of the sponsor, CHU de Nantes. They are not publicly available but are available for scientific review from the corresponding author on reasonable request, under the conditions defined by the General Data Protection Regulation (GDPR) and the CNIL methodology of reference MR004 and after removal of commercially confidential information. Before any data access, a data transfer agreement shall be signed with the sponsor defining the scope of the data transfer, as required by the GDPR, and including an obligation to use the data for the sole purpose of the scientific review and forbidding their disclosure to third parties.
